# Triple-Cation Perovskite Photoanodes for Solar Water Splitting: From Photovoltaic-Assisted to Immersed Photoelectrochemical Operation

**DOI:** 10.3390/mi17040431

**Published:** 2026-03-31

**Authors:** Vera La Ferrara, Marco Martino, Antonio Marino, Giovanni Landi, Silvano Del Gobbo, Nicola Lisi, Rosanna Viscardi, Alberto Giaconia, Giulia Monteleone

**Affiliations:** 1 Energy Technologies and Renewable Sources Department, ENEA Portici Research Center, 80055 Portici, Italy; marco.martino@enea.it (M.M.); antonio.marino@enea.it (A.M.); 2 Energy Efficiency Unit Department, ENEA Portici Research Center, 80055 Portici, Italy; giovanni.landi@enea.it; 3 Energy Technologies and Renewable Sources Department, ENEA Casaccia Research Center, 00123 Rome, Italy; silvano.delgobbo@enea.it (S.D.G.); nicola.lisi@enea.it (N.L.); rosanna.viscardi@enea.it (R.V.); alberto.giaconia@enea.it (A.G.); giulia.monteleone@enea.it (G.M.)

**Keywords:** perovskite photoanode, solar water splitting, hydrogen evolution, encapsulation, ABPE, mixed-halide perovskite, photoelectrochemical devices, PET–EVA

## Abstract

Mixed-halide perovskite solar cells with the composition Cs_0.1_(MA_0.17_FA_0.83_)_0.9_Pb(I_0.83_Br_0.17_)_3_ were fabricated obtaining solar cells as glass/ITO/SnO_2_/triple-cation perovskite/HTL/Au, and subsequently used as photoanodes for efficient solar-driven water splitting by attaching commercial catalytic nickel foils to the Au back-contact pads of solar cells. To enable operation in alkaline media, the devices were encapsulated using commercial PET–EVA multilayer films, providing an effective barrier while leaving the Ni foils exposed as the electrochemically active interface. Two operating configurations were investigated and compared: (i) an outside configuration, where the perovskite device powered the external electrochemical cell, and (ii) an immersed configuration, in which the encapsulated perovskite solar cell was directly integrated, together with the Ni catalyst, into the electrolyte. In both configurations, the onset potential for the oxygen evolution reaction shifted from ~1.32 V vs. RHE, when the Ni electrode was not powered by the perovskite solar cell, to ~0.34 V vs. RHE, when the perovskite device powered the Ni foil for both immersed and outside configurations. The immersed configuration delivered the highest performance, achieving a maximum Applied Bias Photon-to-Current Efficiency of ~20% under AM 1.5 G illumination (100 mW cm^−2^), among the highest values reported for perovskite-based photoanodes. Importantly, the enhanced performance does not arise from changes in catalyst composition or direct semiconductor–electrolyte interaction, but from improved photovoltage delivery and reduced resistive losses enabled by the integrated device architecture. These results demonstrate that device architecture is a key factor in controlling photovoltage utilization and charge-transfer kinetics, providing a viable strategy for efficient and scalable perovskite-based photoelectrochemical systems.

## 1. Introduction

Organic–inorganic hybrid perovskite solar cells (PSCs) have attracted remarkable attention as next-generation photovoltaic materials due to their high-power conversion efficiencies, low-cost fabrication, and tunable optoelectronic properties [1,2,3]. Despite these advantages, most perovskite devices suffer from rapid degradation upon exposure to moisture, oxygen, and light, which severely limits their operational stability and long-term performance [4]. This challenge underscores the need for innovative encapsulation strategies and device integration that can simultaneously ensure high efficiency and environmental robustness. These intrinsic vulnerabilities remain one of the main obstacles to the commercialization of perovskite-based technologies. To enhance device durability, several strategies have been proposed, including compositional engineering, interface passivation, and the use of flexible polymeric encapsulants [5,6,7,8,9]. Polymeric barriers improve resistance to humidity and oxygen while enabling lightweight, robust, and portable devices compared to heavier commercial alternatives. These encapsulation approaches allow perovskite-based systems to operate in conditions unsuitable for unprotected devices [10]. Encapsulation also enables the integration of PSCs with photoelectrochemical (PEC) systems for solar-driven water splitting, where protection from aqueous environment is essential. Such integrated configurations allow hydrogen production directly from sunlight while avoiding both the intrinsic instability of perovskites in liquid electrolytes and the need for an external power supply. Integrating the light absorber and catalytic electrodes within a compact architecture simplifies the device design, reduces energy losses, and potentially improves conversion efficiency. The adoption of a PEC approach enables the direct conversion of solar energy into chemical fuels, offering a pathway toward integrated Solar-to-Hydrogen systems. This approach has been widely recognized as a promising route toward sustainable hydrogen production, as it allows direct coupling between light absorption and catalytic reactions without intermediate electrical conversion steps [11]. In solar fuel research, water splitting can be achieved through either photocatalytic or photoelectrochemical (PEC) approaches. In photocatalysis, light absorption, charge separation, and catalytic reactions occur within suspended semiconductor particles, often making charge extraction and interfacial processes difficult to control. In contrast, PEC systems rely on photoelectrodes connected to an external circuit, enabling spatial separation of redox reactions and direct measurement of photocurrent and applied bias. This configuration provides a more controlled and quantitative platform to investigate charge-transfer kinetics and photovoltage utilization. In this context, the PEC approach was specifically chosen to evaluate how effectively the photovoltage generated by encapsulated perovskite solar cells can be delivered to the catalytic interface and exploited to drive water-splitting reactions under alkaline conditions. This is particularly relevant for understanding the role of device architecture in governing photovoltage delivery, interfacial charge-transfer kinetics and overall device performance. Perovskite-based devices can provide high photovoltages, but their effective utilization strongly depends on the device integration and on how efficiently the photovoltage is transferred to drive interfacial electrochemical reactions. Moreover, the bandgap tunability of hybrid perovskites, combined with their high absorption coefficients, makes them promising photoanode materials capable of driving the oxygen evolution reaction (OER) efficiently under alkaline conditions [12,13,14,15,16].

The use of perovskite materials as photoanodes has gained increasing importance due to their unique combination of optoelectronic and structural properties that can overcome the limitations of conventional metal oxides. Traditional metal oxide-based photoanodes such as BiVO_4_, WO_3_, and Fe_2_O_3_ exhibit high stability but suffer from wide bandgaps and sluggish charge transport. In contrast, halide perovskites exhibit high absorption coefficients, long carrier diffusion lengths, and tunable bandgaps (1.5–2.2 eV), making them suitable for solar water splitting [12]. Recent studies have demonstrated that perovskite-based photoanodes can achieve outstanding photoelectrocatalytic activity when properly protected and integrated with efficient catalysts. For instance, Hansora et al. [13] reported FAPbI_3_/NiFeOOH photoanode with Applied Bias Photon-to-Current Efficiency (ABPE) of 7.9%. Similarly, Yang et al. [14] demonstrated that perovskite with carbon/graphite conductive protection layers and NiFe catalyst can achieve unassisted water splitting with a high ABPE of 8.5%. More recently, Fehr et al. [15] demonstrated scalable perovskite PEC systems with Solar-to-Hydrogen (STH) efficiencies of up to 20.8% in tandem configurations, while Song et al. [16] reported all-perovskite tandem systems with STH efficiencies up to 15% and operational stability exceeding 120 h. These advances highlight the capability of perovskite materials to deliver high photovoltage (>2.0 V) and high photocurrent densities (>12 mA cm^−2^), while maintaining low fabrication costs. However, their integration into aqueous PEC environments remains challenging. Direct exposure to electrolytes often results in structural degradation and performance loss due to ion migration and dissolution of the perovskite layer. Therefore, the development of encapsulation approaches that combine environmental stability with efficient electronic coupling to catalytic electrodes is critical for advancing this technology. Among the various perovskite compositions, triple-cation mixed-halide systems (e.g., Cs/MA/FA-based) have emerged as particularly suitable for applications requiring enhanced operational stability. Compared to single- or double-cation perovskites, triple-cation formulations exhibit improved phase stability, reduced defect density, and suppressed ion migration, leading to enhanced charge transport and reduced non-radiative recombination losses. These effects were systematically demonstrated by Saliba et al. [17], who showed that the incorporation of cesium into mixed-cation perovskites significantly enhances thermal and structural stability. These advantages are particularly relevant under PEC conditions, where the device is subjected to additional stress factors such as continuous illumination, applied bias, and proximity to aqueous electrolytes. In such environments, less stable perovskite compositions tend to degrade rapidly due to structural instability and interfacial reactions. Therefore, the use of triple-cation perovskites represents a rational and strategic choice to enhance both device durability and performance in PEC configurations. To date, only limited studies have demonstrated their feasibility for water splitting [15] often without a detailed photoelectrochemical characterization or a systematic analysis of device integration. In particular, a direct comparison between perovskite solar cells operating externally to an electrochemical cell and those integrated within the electrolyte has not been reported. As a result, the role of device configuration in governing photovoltage delivery, interfacial charge-transfer kinetics, and overall PEC performance remains unclear. In this context, the present work addresses this gap by providing a systematic comparison between outside (OS) and immersed (IS) configurations using the same encapsulated triple-cation perovskite device. The OS configuration corresponds to a photovoltaic-assisted electrochemical cell setup, whereas the IS configuration involves direct integration of the solar cell-based device, together with the Ni catalyst, into the electrolyte. This approach allows the effect of device architecture to be isolated. Furthermore, we demonstrate that the enhancement in PEC performance does not originate from changes in catalyst composition or direct semiconductor–electrolyte interaction, but rather from improved photovoltage delivery and reduced resistive losses enabled by the integrated device. Finally, this work provides one of the first comprehensive photoelectrochemical characterizations of triple-cation perovskites under alkaline conditions, including cyclic voltammetry, onset potential analysis, photocurrent response, and ABPE evaluation. In this work, we employ a triple-cation mixed-halide perovskite, Cs_0.1_(MA_0.17_FA_0.83_)_0.9_Pb(I_0.83_Br_0.17_)_3_, as a light absorber layer in n-i-p solar cells, glass/ITO/SnO_2_/triple-cation perovskite/HTL/Au and subsequently adapted to operate as photoanodes. This formulation of triple-cation perovskite, with a higher cesium content and optimized halide ratio, enhances structural stability and interfacial charge transfer [17,18]. To enable operation in alkaline electrolyte, the devices were encapsulated using commercially available PET–EVA multilayer films, forming the final architecture glass/ITO/SnO_2_/perovskite/HTL/Au/Ni, where Ni acts as the catalytic interface.

## 2. Materials and Methods

### 2.1. Materials

SnO_2_ colloid precursor (tin (IV) oxide 15% H_2_O colloidal dispersion), PbI_2_ (≥99.999%, ultradry) and PbBr_2_ (Puratronic, ≥99.998%) were purchased from Alfa Aesar (Thermo Fisher Scientific, Rodano, Italy). CsI (≥99.999%, anhydrous), acetonitrile (ACN anhydrous), anisole (≥99.7% anhydrous), N,N-dimethylformamide (DMF anhydrous), dimethyl sulfoxide (DMSO anhydrous), formamidinium iodide (FAI anhydrous), methylammonium bromide (MABr, ≥99%, anhydrous), Spiro-OMeTAD, chlorobenzene (CB anhydrous), 4-tert-butylpyridine (TBPy), bis(trifluoromethane)sulfonimide lithium salt (Li-TFSI), and FK209 Co (III) TFSI salt were purchased from Sigma Aldrich (Merck Life Science S.r.l., Milano, Italy). All chemicals were used without further purification. Glass/ITO substrates (2 × 2 cm^2^, 10 Ω sq^−1^) were used as conductive supports and purchased from Kintec (Hong Kong, China).

### 2.2. Preparation of Precursors

The SnO_2_ solution was prepared by diluting the commercial SnO_2_ colloidal dispersion with deionized water (1:5). The perovskite precursor solution Cs_0.1_(MA_0.17_FA_0.83_)_0.9_Pb(I_0.83_Br_0.17_)_3_ was obtained by mixing PbI_2_ (1.10 M), PbBr_2_ (0.22 M), FAI (1.05 M), and MABr (0.20 M) in DMF/DMSO (4:1 v/v). A 1.50 M CsI solution in DMSO was added to achieve 10 mol% Cs content. The hole transport material (HTM) solution consisted of 73 mg Spiro-OMeTAD in 1 mL CB, 27 µL TBPy, 17 µL Li-TFSI solution (520 mg Li-TFSI in 1 mL ACN), and 7 µL FK209 Co (III) TFSI solution (375 mg in 1 mL ACN). The alkaline electrolyte (1 M KOH) was prepared by dissolving 5.61 g KOH pellets (≥99.99%), purchased from Sigma Aldrich (Merck Life Science S.r.l., Milano, Italy) in 100 mL deionized water with stirring, cooled to room temperature, and freshly used.

### 2.3. Perovskite-Based Solar Cell and Photoanode Fabrication

**Figure 1:**
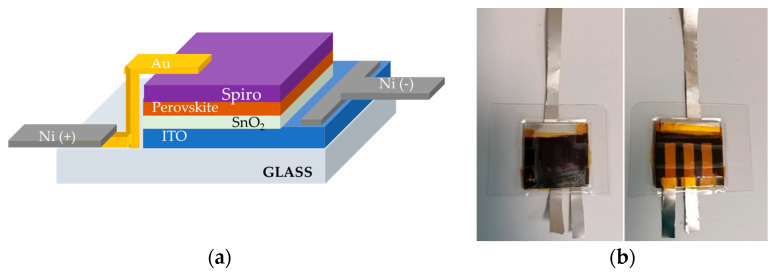
(**a**) Schematic representation of the n-i-p perovskite solar cell architecture (Glass/ITO/SnO_2_/Perovskite/Spiro-OMeTAD/Au) showing the placement of nickel electrodes in contact with Au and ITO terminals of the device. (**b**) Photographs of the typical perovskite device encapsulated using multilayer polymeric films (PET–EVA). (Left) front, where glass is illuminated by solar simulator, and (right) back, where Nickel foils are deposited on Au pads and on ITO.

ITO substrates were sequentially sonicated in acetone and 2-propanol for 15 min each, dried with nitrogen, and treated under UV–ozone for 20 min. SnO_2_ electron transport layers (ETLs) were spin-coated at 6000 rpm for 30 s and annealed at 130 °C for 1 h. The substrates were then transferred into a nitrogen glovebox for perovskite and hole transport layer (HTL) deposition. The perovskite layer was spin-coated using a two-step program (1000 rpm for 10 s and 6000 rpm for 20 s), with 200 µL anisole added dropwise as an antisolvent. Films were annealed at 100 °C for 1 h. The Spiro-OMeTAD layer, as HTL, was spin-coated at 4000 rpm for 30 s, followed by thermal evaporation of 80 nm Au back contact [16]. To use the pristine perovskite solar cell as a photoanode for PEC testing, the device was modified by adding two nickel foils onto its existing electrical contacts, one on the Au pad and one on the ITO-coated glass substrate, named Ni (+) and Ni (−) respectively (Figure 1a). Given the higher rigidity and hardness of nickel compared to gold, small drops of silver conductive paste were applied between the Au pads (or ITO) and the Ni foils to improve electrical contact. To evaluate a suitable thickness of nickel foils for photoelectrocatalytic integration, the current density–voltage (J–V) characteristics of pristine perovskite devices were first recorded under standard illumination (AM 1.5 G, 100 mW cm^−2^) and compared with PSCs with nickel foils of varying thicknesses as the electrode. Among the thicknesses, 20 µm thick nickel foil exhibited the best performance. The perovskite devices were then encapsulated using multilayer polymeric film lamination between two PET–EVA sheets and laminated using a hot-press laminator (PlastiEVO, Tosingraf S.r.l., Rosà, Italy). The lamination process was carried out at 100 °C for 5 min, forming an effective barrier against moisture and oxygen. The PET layer provided mechanical stability and optical transparency, whereas the EVA layer ensured strong adhesion and air- and watertight sealing. A Kapton polyimide tape was first applied to protect the active area from direct exposure to the encapsulant. Front- and back-view images of the encapsulated devices are shown (Figure 1b).

## 3. Results and Discussion

### 3.1. Perovskite-Based PEC Configurations and Characterization of Standard Electrochemical Cell

**Figure 2:**
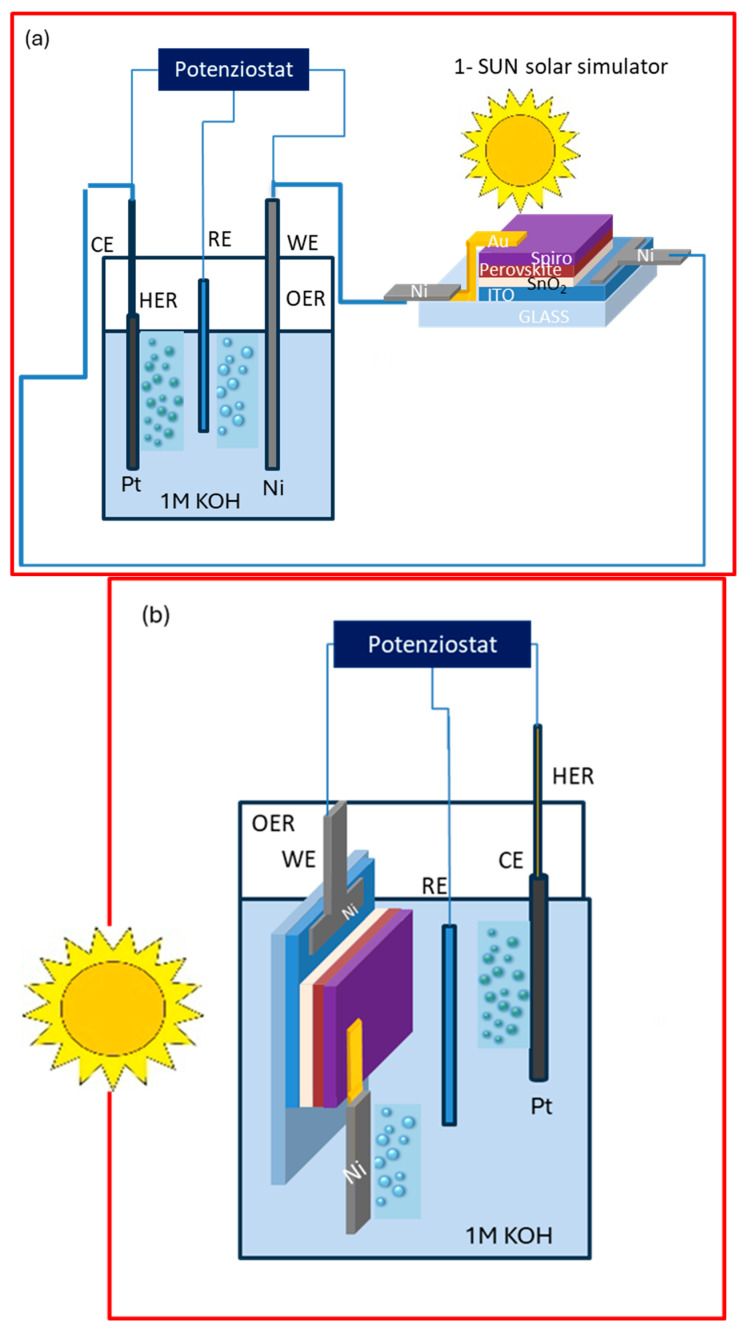
Schematic representation of the encapsulated PSC in (**a**) outside and (**b**) immersed configuration with Nickel as the working electrode and platinum as the counter electrode and the third electrode as reference.

**Figure 3:**
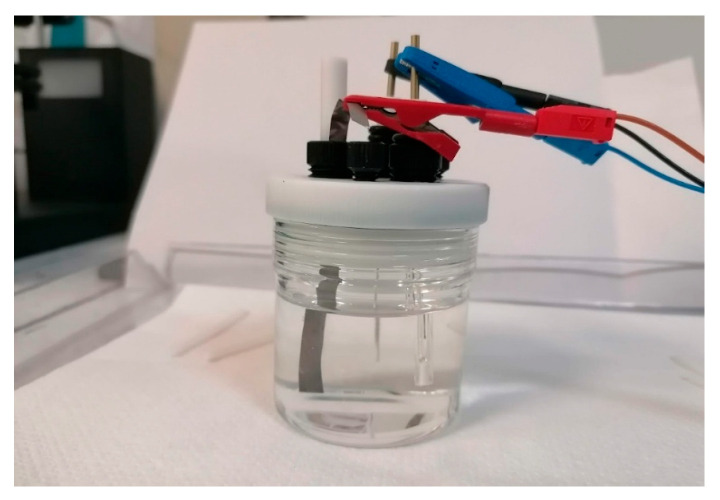
Standard photoelectrochemical cell used for water-splitting experiments in 1 M KOH aqueous electrolyte. The electrodes are biased to the potentiostat via alligator clips.

The photoelectrochemical performance of the encapsulated PSCs was evaluated in two operational configurations: (i) outside (OS) configuration, where the perovskite cell powered an external standard electrochemical cell, and (ii) immersed (IS) configuration, where the encapsulated device was directly integrated into the electrolyte. The Ni foil in contact with the Au terminal was used as the working electrode. Illumination was provided by a LED AM 1.5 G solar simulator (Ossila B.V. Leiden, Netherlands), irradiance 100 mW cm^−2^ (Figure 2). The standard electrochemical had three-electrode configuration with 1 M KOH electrolyte, with Ni as the working electrode, Pt as the counter electrode, and Ag/AgCl as reference (Figure 3). The nickel electrode drove the oxygen evolution reaction (OER) at the anode (4OH^−^ → O_2_ + 2H_2_O + 4e^−^), while the platinum counter electrode catalyzed the hydrogen evolution reaction (HER) at the cathode (4H_2_O + 4e^−^ → 2H_2_ + 4OH^−^). The overall process corresponded to water splitting (2H_2_O → 2H_2_ + O_2_). The cyclic voltammetry (CV) recorded on the Ni/Pt electrodes, biased with a potentiostat, but in the absence of the perovskite solar cell (i.e., without any photovoltage contribution) shows the characteristic behavior of alkaline OER, with an anodic onset potential appearing at approximately E_(onset (vs. Ag/AgCl))_ = +0.30 V vs. Ag/AgCl. The onset potential (E_onset_) is conventionally defined as the potential at which the photocurrent density reaches a small but measurable value (typically 0.0001–0.0002 A cm^−2^ above the baseline), and represents the minimum external bias required to initiate the electrochemical reaction.

To demonstrate the analyzed results, the measured potentials are converted into potential versus reversible hydrogen electrode (RHE) using [19]

(1) $$E_{RHE}=E_{Ag/AgCl}+E_{Ag/AgCl}^{0}+0.059\times pH$$

**Figure 4:**
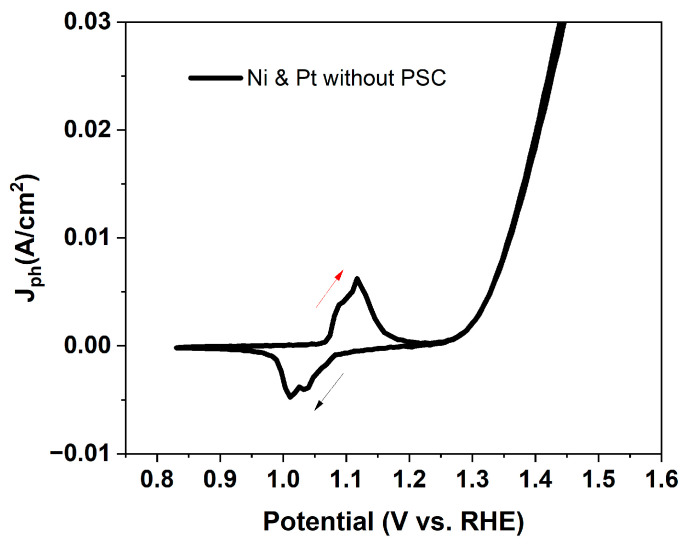
Cyclic voltammetry curve of a standard electrochemical cell, biased with a potentiostat, employing nickel as the working electrode and platinum as the counter electrode in 1 M KOH aqueous solution, at scan rate 100 mV/s. The arrows indicate the direction: from positive to negative voltages (dark arrow, reduction) and from negative to positive voltages (red arrow, oxidation).

The potential of the reference electrode, concerning the standard hydrogen electrode at 25 °C (0.197 V for Ag/AgCl), is denoted as $E_{Ag/AgCl}^{0}$, while the working potential measured versus the reference electrode is represented as $E_{Ag/AgCl}$. Thus, the OER onset corresponds to approximately 1.32 V vs. RHE for nickel-based catalysts in alkaline media (1 M KOH, pH = 14) (Figure 4). The Ni(OH)_2_/NiOOH redox transition, which reflects the formation of active NiOOH sites, occurs at a lower potential of around 1.1 V vs. RHE.

### 3.2. Encapsulated Device Characterization for PEC Applications

**Figure 5:**
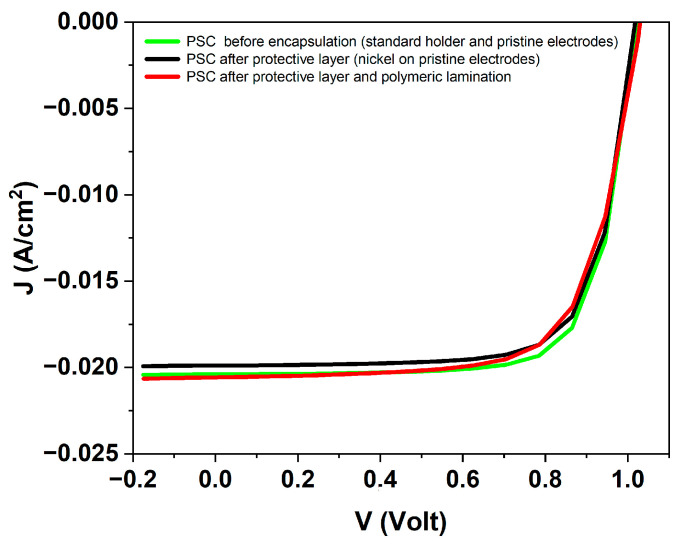
Typical J–V characteristics of PSCs recorded before and after encapsulation. The green curve corresponds to the pristine device measured in the standard holder with original electrodes (gold as positive and ITO as negative electrode); the black curve represents the device after applying the protective Kapton layer and catalytic nickel foil electrodes on the pristine contacts; and the red curve shows the device after full encapsulation with the polymeric multilayer lamination (PET–EVA).

**Figure 6:**
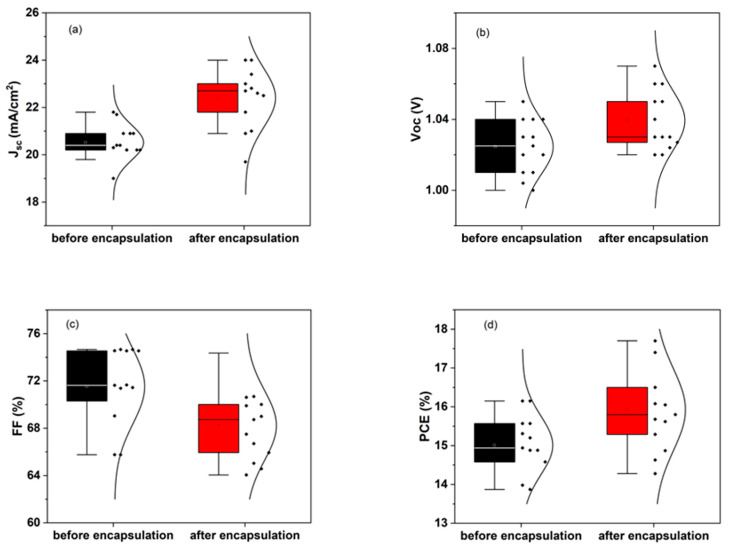
Statistical distribution of photovoltaic parameters before and after encapsulation: (**a**) short-circuit current density (J_sc_), (**b**) open-circuit voltage (V_oc_), (**c**) fill factor (FF) and (**d**) power conversion efficiency (PCE).

After fabrication of PSCs, the J–V characteristics under LED solar simulator, recorded before and after encapsulation, are shown in Figure 5. The nearly overlapping curves confirm that the encapsulation processes do not affect the electrical performance of the devices. To assess whether the encapsulation procedure alters the photovoltaic performance of the devices, a statistical comparison of the key photovoltaic (PV) parameters (short-circuit current density, J_sc_, open-circuit voltage, V_oc_, fill factor, FF, and power conversion efficiency, PCE) was carried out before and after encapsulation. Figure 6 shows the distribution of each parameter across multiple devices, represented as box-and-whisker plots with the individual datapoints overlaid. This analysis allows a direct evaluation of potential degradation or variability induced by the encapsulation process.

The distributions of PV parameters reveal that encapsulation does not negatively affect the photovoltaic performance of the PSCs. A slight increase in J_sc_ is observed after encapsulation. This improvement is attributed to a reduction in surface and interfacial recombination losses. In particular, the encapsulation layers act as an effective barrier against moisture and oxygen, which are known to induce trap states and accelerate interfacial degradation in perovskite devices. In addition, the lamination process can improve interfacial contact between layers, particularly at the back contact, thereby facilitating more efficient charge extraction. A minor contribution from enhanced optical confinement within the encapsulated structure may also play a role. Similar trends have been reported in the literature, where encapsulation strategies were shown not only to enhance environmental stability, but also to suppress non-radiative recombination and ion migration at perovskite interfaces, leading to improved photovoltaic performance, although different encapsulation chemistries were used [20]. Overall, these effects contribute to more efficient carrier collection, resulting in the observed increase in photocurrent. These results confirm the robustness of the encapsulation strategy and demonstrate that the devices retain full photovoltaic functionality prior to their implementation as photoanodes in PEC measurements.

### 3.3. Experimental Setup for Perovskite Solar Cells in Outside and Immersed Configurations

The photoelectrochemical performance of the encapsulated PSCs (called PSC A, PSC B, PSC C and PSC D) was evaluated in two operational configurations: outside and immersed.

#### 3.3.1. Outside Configuration

**Figure 7:**
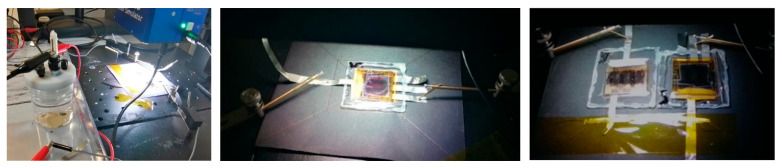
Photographs of the experimental setup used for testing PSCs in outside configuration under LED solar illumination. (**Left**) The encapsulated PSC is connected externally to the standard electrochemical cell. (**Center**) Single perovskite solar cell. (**Right**) Two PSCs connected in series.

In the OS configuration, the encapsulated PSCs operated as independent light-harvesting units, directly powering the external electrochemical cell for water splitting. Figure 7 shows the corresponding photographs. The left panel displays the full experimental setup, including the electrochemical cell, the solar simulator, and the microprobes used to contact the external perovskite solar cell. In the central image, a single external PSC is contacted with two microprobes, while the right image shows two PSCs connected in series and contacted in the same manner, both illuminated under identical conditions.

**Figure 8:**
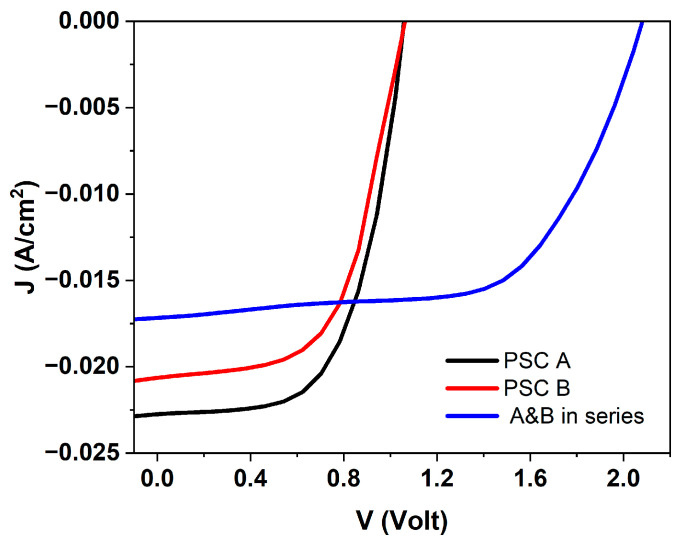
Typical current density–voltage (J–V) characteristics of one PSC and of the two devices connected in series, lighted with LED solar simulator.

**Figure 9:**
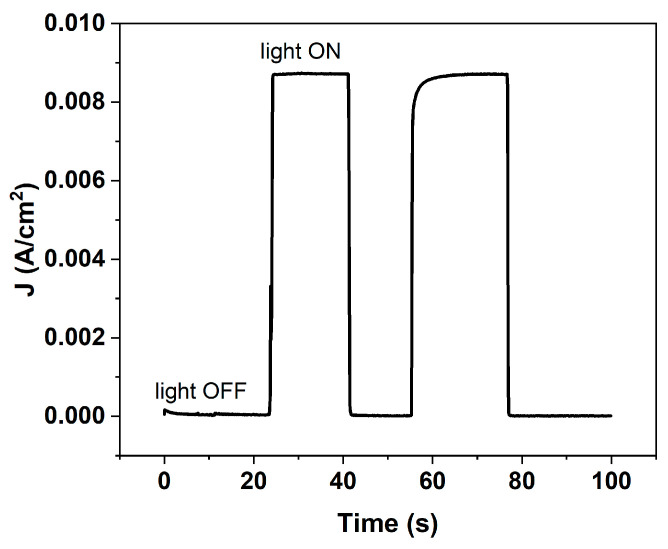
Photocurrent density of the photoelectrochemical cell connected to PSCs in series, lighted by LED solar simulator and no bias with potentiostat.

Typical J–V curves (Figure 8) confirm that connecting two PSCs in series increases the open-circuit voltage (V_oc_) as expected, while the current density (J) decreases slightly due to additional contact resistance introduced by the series connection. In the OS configuration, the only driving force for the reaction was the photovoltage delivered by the perovskite solar cell placed outside the electrolyte, which powered the Ni/Pt electrochemical cell. All measurements in alkaline electrolyte were performed under zero applied bias from the potentiostat. No hydrogen evolution was detected in the dark, confirming the absence of spontaneous electrochemical activity. Under illumination, the photocurrent was observed, demonstrating that the photovoltage generated by external PSCs was sufficient to autonomously power the water-splitting reaction. The chronoamperometric response (Figure 9) clearly shows a marked increase in photocurrent (J_ph_) upon illumination, corresponding to hydrogen generation, and a return to baseline in the dark.

#### 3.3.2. Immersed Configuration

**Figure 10:**
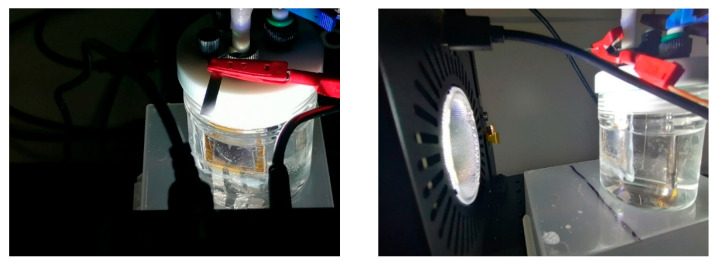
Photographs of the photoelectrochemical setup when perovskite-based photoanode was immersed. (**Left**) The encapsulated perovskite-based photoanode illuminated through the glass substrate using the LED solar simulator. (**Right**) Another view of the same setup during operation.

**Figure 11:**
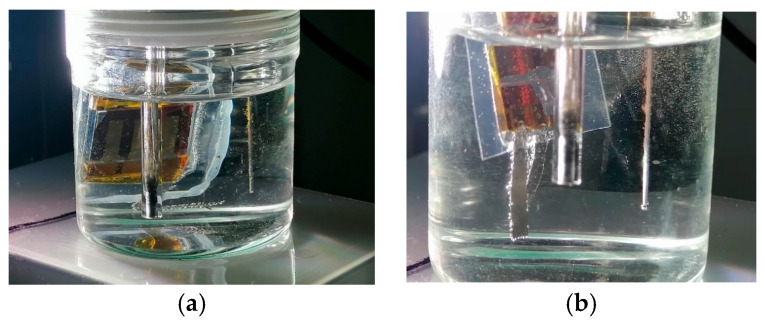
Magnified views of the perovskite-based photoanode showing the actual placement of the nickel electrodes on the Au terminals after encapsulation. (**a**) Configuration with a single Ni electrode placed on one Au pad of the device. (**b**) Configuration with two Ni electrodes positioned on two separate Au pads, defining independent photoactive areas sharing a common ITO contact.

**Figure 12:**
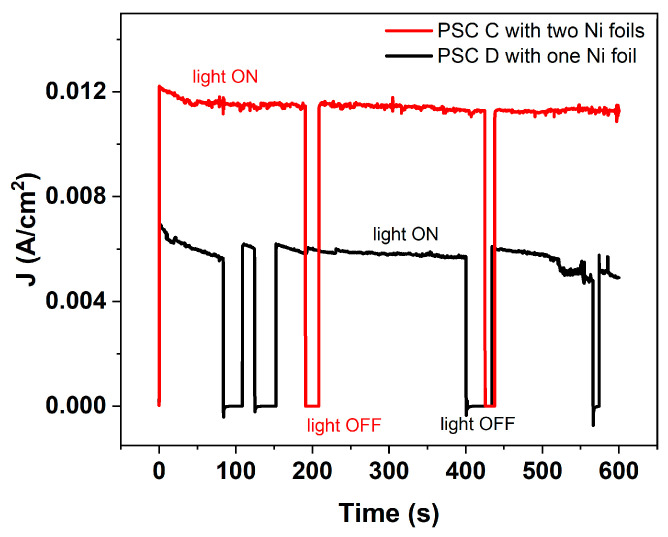
Photocurrent–time (J_ph_–t) response of photoanode under illumination but no bias with potentiostat. The black curve corresponds to a device with a single Ni foil electrode placed on one Au pad, while the red curve refers to a device with two Ni foil electrodes positioned on two separate Au pads. PSC C-Ni foil 0.56 cm^2^ each, PSC D-Ni foil 0.6 cm^2^.

In the IS configuration, the encapsulated perovskite solar cell was directly placed in the alkaline electrolyte, serving simultaneously as the photovoltaic absorber and as the mechanical support for the nickel electrode that defined the active area, and no bias is applied from the potentiostat. In this arrangement, the PSC was fully integrated within the electrochemical environment. Photographs of the experimental setup are presented in Figure 10 and Figure 11, where LED solar simulator lighted through the substrate glass side. The light beam was oriented perpendicularly to the photoanode surface to ensure uniform illumination of the active area. Figure 12 compares the transient photocurrent generated (J_ph_) by one PSC when contacted with either one or two nickel foils. When two Ni foils are connected to the two Au pads of the PSC (red curve), the photocurrent nearly doubles relative to the configuration with a single Ni foil (black curve). This increase is consistent with the increasing of the electrochemically active area while the operating voltage remains unchanged, confirming that the photocurrent scales proportionally with the area of active Ni contact interfaces participating in the OER process. The transient J_ph_–t profiles exhibited clear ON/OFF photoresponses synchronized with light modulation, confirming that no reaction occurred in the dark and unbiased (see Videos S1 and S2 recorded with a single nickel foil and two nickel foils, respectively).

**Figure 13:**
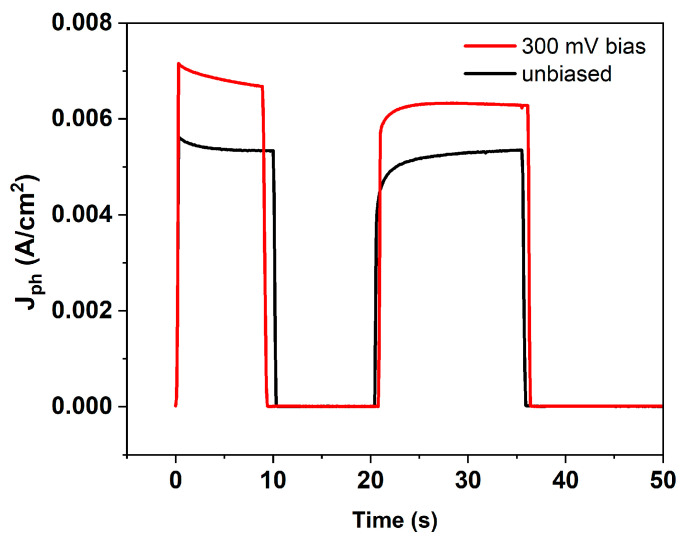
Photocurrent–time response of the immersed perovskite photoanode under chopped illumination. The black curve corresponds to measurements performed at 0 V bias, while the red curve represents data acquired under an applied bias of +300 mV. PSC had 3 Nickel foils, 0.5 cm^2^ each.

The influence of externally applied bias from the potentiostat on the PEC performance was also examined (Figure 13). As expected, the photocurrent increased if 300 mV bias was applied, indicating improved charge separation and enhanced interfacial charge-transfer kinetics. No measurable current or hydrogen evolution was observed in the dark, even under applied bias, confirming the strictly photoinduced nature of the process.

### 3.4. Photoelectrochemical Response: Outside vs. Immersed Configuration

**Figure 14:**
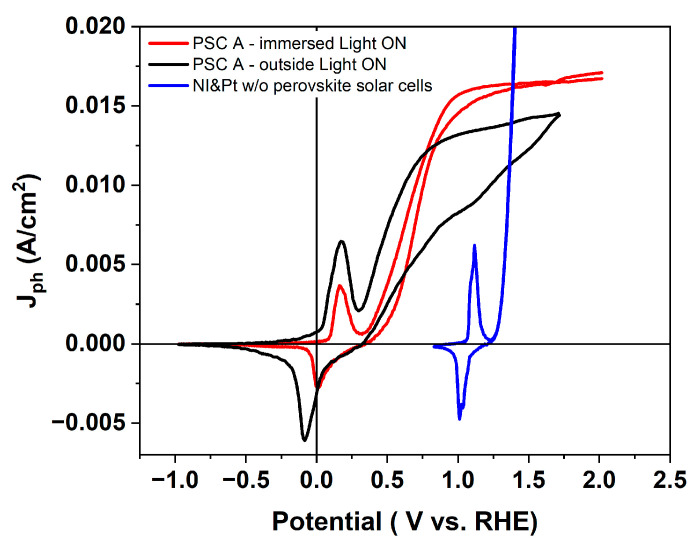
Cyclic voltammetry curves of the encapsulated perovskite in both OS and IS configurations, dark and red curves respectively, compared to the cyclic voltammetry of a standard electrochemical cell (the blue one).

A comparative CV analysis was performed on the same encapsulated PSC, first operated outside the electrolyte and then immersed in the electrolyte to enable a direct comparison. The measurements were also compared with those of a standard Ni electrode measured without the perovskite solar cell. (Figure 14). The CV curves were recorded without iR compensation; however, the series resistance of the system (4–7 Ω) results in a negligible uncompensated drop, making this correction unnecessary for interpreting the data. Both configurations, OS and IS, exhibit hysteresis between the reduction and oxidation scans, a characteristic of perovskite-based photoelectrodes associated with ionic migration and capacitive charging within the halide perovskite absorber [21]. However, hysteresis is noticeably more pronounced in the OS configuration (dark curve Figure 14), indicating slower interfacial equilibration and higher recombination losses when the device is electrically connected through external wiring. In contrast, the immersed device shows a more stable and reproducible CV shape, suggesting improved charge-transfer kinetics. This difference can be rationalized considering the electrical and electrochemical coupling in the two configurations. In the OS configuration, the photovoltage generated by the perovskite is delivered through external wiring and metallic contacts before reaching the electrolyte, introducing additional series resistance, contact losses, and potential drops at multiple interfaces. These effects reduce the effective driving force available for the electrochemical reaction and slow down interfacial charge transfer. In contrast, in the IS configuration, the catalyst is directly integrated within the electrochemical environment and electrically coupled to the perovskite back contact, minimizing resistive losses and enabling a more efficient transfer of photogenerated holes to the NiOOH active sites. This improved coupling enhances the kinetics of the Ni(OH)_2_/NiOOH redox transition and facilitates faster charge injection into the electrolyte. These effects contribute to the observed history-dependent electrochemical response, which is absent in the standard electrochemical cell where the Ni and Pt electrodes are not powered by the perovskite solar cell (blue curve; Figure 14). Moreover, in the IS configuration (red curve; Figure 14), the photocurrent increases more rapidly and reaches higher values compared to the OS configuration. The onset potential for oxygen evolution shifts dramatically when the PSC is used for powering the nickel foil electrode for both IS and OS configurations. While the Ni/Pt electrochemical cell, not powered by the perovskite absorber, exhibits an onset around 1.32 V vs. RHE (Figure 14; blue curve), both the IS and OS PSC–Ni configurations show a much earlier onset at ~0.34 V vs. RHE (Figure 14; black and red curves).

This strong reduction in overpotential does not originate from the Ni–electrolyte interface itself, as nickel already forms Ni(OH)_2_/NiOOH in the standard cell, but rather from the photovoltage and photogenerated holes supplied by the perovskite absorber directly into the NiOOH catalytic sites, triggering the Ni(OH)_2_/NiOOH redox transition at approximately 177 mV (Figure 14; black and red curves). This value is markedly lower than the anodic peak observed at ~1.1 V in the configuration where the electrochemical cell operates without being powered by the PSC (Figure 14; blue curve). This comparison clearly shows that the photovoltage provided by the perovskite significantly lowers the energetic threshold required for Ni activation in alkaline media. This behavior indicates that the photovoltage generated by the perovskite effectively shifts the Fermi level of the electrode under illumination, enabling earlier activation of the catalytic cycle. In this sense, the perovskite does not merely act as a power source but actively modulates the electrochemical potential at the catalyst interface. Furthermore, the IS configuration shows the highest photocurrents and the most favorable kinetics, whereas the OS configuration still benefits from photovoltage assistance but is limited by additional resistive and interfacial losses. Taken together, the comparison demonstrates that the perovskite solar cell is responsible for the drastic reduction in OER onset potential and that direct immersion maximizes the efficiency of photovoltage transfer to the catalytic interface and reduces hysteresis compared to the OS configuration. Overall, these results demonstrate that the observed enhancement in the IS configuration is not due to changes in catalyst composition or intrinsic activity, but rather to improved photovoltage delivery and reduced resistive losses enabled by the integrated device architecture. This highlights the critical role of device design in maximizing the utilization of photogenerated charge carriers in perovskite-based PEC systems. Although the CV curves highlight clear differences in onset potential and photocurrent behavior between the two configurations, a quantitative metric is needed to determine how effectively the devices convert absorbed photons into chemical output.

Although direct quantification of H_2_/O_2_ and Faradaic efficiency was beyond the scope of the present study, these measurements will be necessary in future work to validate practical energy conversion and determine the corresponding Solar-to-Hydrogen (STH) efficiency. Therefore, in this study, the Applied Bias Photon-to-Current Efficiency (ABPE) was used as a direct and reliable metric of photoelectrochemical performance.

**Figure 15:**
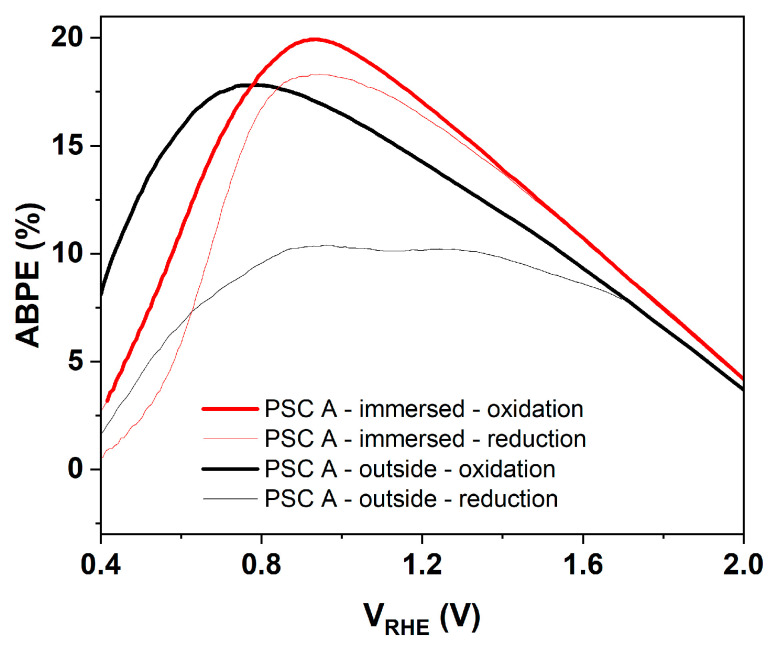
Applied Bias Photon-to-Current Efficiency curves for the PSC operated in the OS and IS configurations.

The ABPE is defined as

(2) $$ABPE(\%)=\frac{J_{ph}\times (1.23-V_{bias})}{P_{in}}\times 100$$

where J_ph_ is the photocurrent density (A cm^−2^), 1.23 V is the thermodynamic potential required for water splitting, V_bias_ is the applied bias potential, and P_in_ is the incident light power density (100 mW cm^−2^ under standard AM 1.5 G solar illumination). The potential corresponding to the maximum of the ABPE curve is referred to as the optimal bias (V_opt_), which represents the point of maximum conversion efficiency (Figure 15).

The IS configuration delivers the highest ABPE (~19.9%) because it minimizes contact resistance and enables more efficient photovoltage transfer to the Ni/NiOOH interface, whereas the OS configuration (~17.8%) remains limited by additional wiring and interfacial losses. Table 1 summarizes the photoelectrochemical parameters obtained for both the IS and OS configurations.

**Figure 16:**
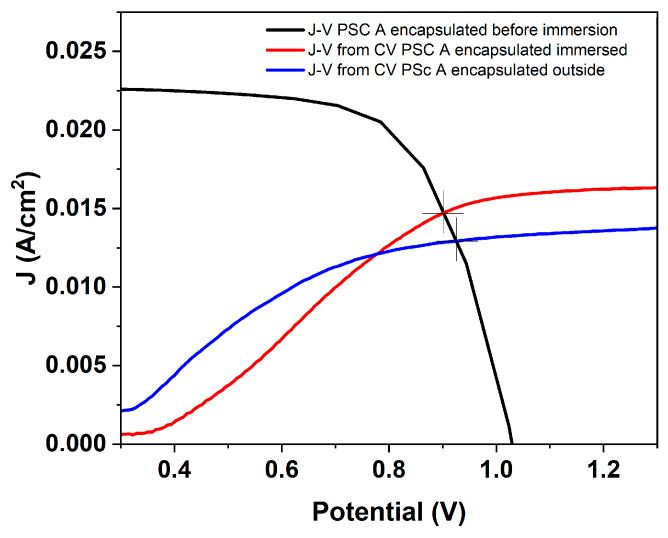
J–V curve of the perovskite solar cell (black, before immersion) overlaid with the photocurrent–potential responses extracted from CV in the IS (red) and OS (blue) configurations. The intersection point indicates the self-driven operating condition for OER without external bias.

To examine the effective operating conditions of the device in different configurations, the J–V curve, measured for pristine PSC in photovoltaic (PV) mode (before immersion), was overlaid with the photocurrent–potential characteristics extracted from cyclic voltammetry in the IS and OS configurations (Figure 16). This combined representation directly shows how much of the photovoltage generated by the perovskite can be transferred to the electrochemical reaction under each operating mode.

In PV mode (black curve), the device shows V_oc_ of ~1.05 V and short-circuit current density exceeding 20 mA cm^−2^, representing the maximum power available from the perovskite absorber. When the same device operates as an immersed photoanode (red curve), the anodic photocurrent follows a markedly different J–V trajectory, intersecting the PV curve at approximately 0.89 V and ~14.7 mA cm^−2^. This intersection defines the potential operating point at which the photovoltage generated by the perovskite is sufficient to sustain OER without external bias. The higher current at the intersection reflects the more efficient transfer of photovoltage to the Ni/NiOOH interface in the IS configuration, in accordance with improved charge-transfer kinetics and reduced interfacial losses. In contrast, the OS configuration (blue curve) exhibits lower photocurrent and its intersection with the PV curve occurs at a much smaller current density and at a higher voltage, 0.93 V, closer to V_oc_. This indicates that only a limited fraction of the available photovoltage is effectively transferred to the electrochemical cell when the device is not immersed, due to additional series resistance and wiring losses, and the absence of direct semiconductor–electrolyte coupling. As a result, the OS configuration operates close to the OER onset, which explains why only a few high-performing cells can drive oxygen evolution in this configuration.

### 3.5. Stability of Immersed Device

**Figure 17:**
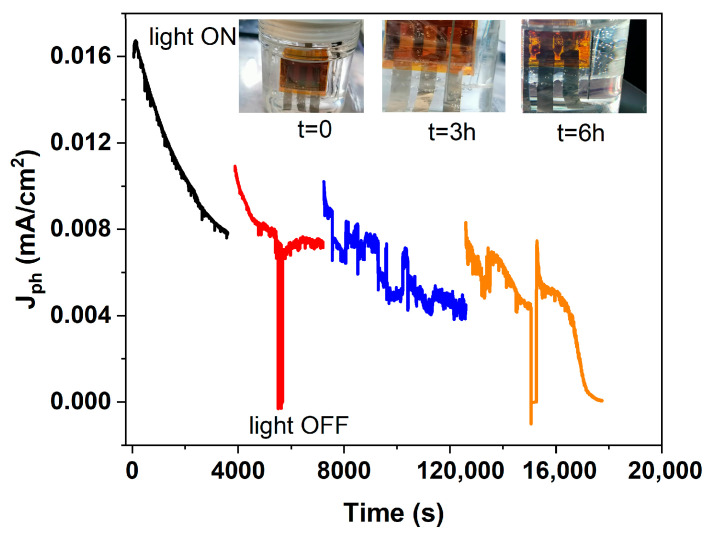
Photocurrent stability of the immersed perovskite-based photoanode under continuous illumination in 1 M KOH. The photocurrent density (J_ph_) is shown as a function of time, with different colors corresponding to consecutive measurement intervals. Photographs of the device at t = 0 h, 3 h, and 6 h during immersion are reported above the graph, highlighting the physical evolution of the sample over time.

To evaluate the operational long-term stability of the immersed perovskite-based photoanode, an aging test was carried out under continuous illumination with an LED solar simulator in 1 M KOH, with simultaneous monitoring of the photocurrent and visual inspection of the device over time (Figure 17).

At t = 0 h, the photograph (inset of Figure 17) shows the device at the beginning of the immersion. After ~3 h of immersion, a yellowish coloration becomes visible along the edges of the encapsulation, indicating that although the PET–EVA lamination provides a good barrier against moisture and alkaline exposure, it is insufficient on its own to fully protect the device edges from electrolyte penetration. Additionally, epoxy resin was applied at the borders to reinforce edge sealing, but partial penetration still occurred during prolonged operation. After 6 h, the perovskite layer shows complete degradation, accompanied by a sharp decrease in photocurrent, confirming that device failure occurs once the electrolyte reaches the active layer. The observed edge degradation likely originates from electrolyte penetration through imperfect sealing, leading to perovskite dissolution and ion migration. Future work should explore advanced edge-sealing materials or multilayer encapsulation strategies. Previous studies have shown that PET–EVA encapsulation can offer effective environmental protection in alkaline media [22,23], suggesting that the operational stability observed here could be further extended through improved edge-sealing strategies.

### 3.6. Comparison with Literature-Reported Approaches

Table 2 compares the IS configuration presented in this study with representative perovskite-based photoelectrodes reported in the literature under similar alkaline conditions (1 M KOH, AM 1.5 G illumination). A direct comparison highlights that the present device achieves among the highest ABPE values reported for single photoanode configurations, reaching ~20%, significantly exceeding previously reported values (typically ≤8.5% for standalone perovskite photoanodes [13,14]). It is important to note that higher efficiencies reported in the literature are often achieved in tandem architectures (e.g., perovskite/silicon systems [15]), where additional photovoltage is provided by a second absorber. In contrast, the performance reported here is obtained using a single perovskite-based photoanode, emphasizing the effectiveness of the proposed device architecture in maximizing photovoltage utilization. Compared to previous immersed configurations, the improved performance can be primarily attributed to the combined effect of the triple-cation composition, which enhances electronic quality and stability, and the optimized device integration that minimizes resistive losses and improves charge-transfer kinetics at the catalyst interface. In terms of operational stability, the device exhibits a degradation timescale of approximately 5 h, comparable to other triple-cation perovskite systems [15], while longer stability reported for single-cation systems is generally associated with significantly lower efficiencies. This highlights the current trade-off between performance and durability in perovskite-based PEC systems. Overall, this comparison demonstrates that high PEC performance can be achieved without relying on complex tandem architectures, but rather through optimized device integration and efficient photovoltage utilization.

Further improvements in encapsulation and edge sealing will be required to extend operational lifetimes while maintaining the high efficiency demonstrated here.

## 4. Conclusions

This work presents a systematic comparison between outside (OS) and immersed (IS) configurations of encapsulated triple-cation perovskite solar cells operating as photoanodes for solar-driven water splitting. The OS configuration corresponds to a photovoltaic-assisted electrochemical cell setup, whereas the IS configuration involves direct integration of the solar cell-based device, together with the Ni catalyst, into the electrolyte. The results clearly demonstrate that device architecture plays a decisive role in governing the photoelectrochemical response. In particular, the immersed configuration significantly enhances charge-transfer kinetics, reduces hysteresis, and increases photocurrent density and overall PEC efficiency compared to the outside configuration. A key finding of this study is that the observed performance enhancement does not originate from changes in catalyst composition or from direct semiconductor–electrolyte interaction, as the perovskite absorber remains fully encapsulated. Instead, the improvement arises from more efficient photovoltage delivery and reduced resistive losses enabled by the integrated device architecture. This provides direct experimental evidence that photovoltage utilization, rather than catalyst modification, is a critical factor in determining the performance of perovskite-based PEC systems. Under AM 1.5 G illumination, the immersed configuration achieves an ABPE of approximately 20%, among the highest values reported for perovskite-based photoanodes, while simultaneously reducing the OER onset potential by nearly 1 V compared to a standard Ni/Pt electrochemical system. From a broader perspective, this study introduces three main advances: (i) the first systematic comparison between outside and immersed configurations, enabling isolation of architectural effects; (ii) the identification of photovoltage delivery and resistive losses as the dominant factors governing PEC performance; and (iii) one of the first comprehensive photoelectrochemical characterizations of triple-cation perovskites under alkaline conditions. In addition to these fundamental insights, the proposed architecture outlines a viable route toward scalable PEC systems, based on solution-processed perovskite absorbers, commercially available PET–EVA encapsulation materials, and a simplified monolithic design that minimizes external wiring and associated losses. Future work should focus on improving long-term operational stability through advanced encapsulation and edge-sealing strategies, as well as on direct quantification of hydrogen and oxygen evolution to determine Solar-to-Hydrogen and Faradaic efficiencies. These developments will be essential to translate the demonstrated performance into practical and scalable solar fuel technologies.

## Figures and Tables

**Table 1:** Summary of key photoelectrochemical parameters for the PSC operated in the OS and IS configurations.

Configuration	V_Onset_ (V vs. RHE)	V_opt_ (V vs. RHE)	Jph (mA/cm^2^)	ABPE_max_ (%)
IS (oxidation curve)	0.34	0.93	15.7	~19.93
IS (reduction curve)	0.34	0.93	15.7	~18.31
OS (oxidation curve)	0.34	0.75	13.6	~17.80
OS (reduction curve)	0.34	0.93	9.9	~10.39

**Table 2:** Recent literature data obtained with the best perovskite photoelectrodes in 1 M KOH, illumination 100 mW cm^−2^.

Photoelectrode Configuration	Jph (mA/cm^2^) at 1.23 V vs. RHE	ABPE (%)	STH (%)	Aging	Ref.
Cs_0.1_(MA_0.17_FA_0.83_)_0.9_Pb(I_0.83_Br_0.17_)_3_ immersed photoanode/Nickel foil	16.4	19.9		5 h	This work
MAPbI_3_–BiVO_4_ photoanode tandem–outside PSC	5.01	6.2			[12]
FAPbI_3_/Ni/NiFeOOH—immersed photoanode	22.8		9.8	3 days	[13]
FAPbBr_3_/carbon/graphite/NiFe immersed photoanode	9.12	8.5		100 h	[14]
Cs_0.05_FA_0.85_MA_0.1_Pb(I_0.95_Br_0.05_)_3_ & FA(PbI_3_)_0.97_(MAPbI_3_)_0.03_ immersed photocathode & photoanode	20	13.4 *		5 h	[15]
Tandem perovskite/silicon phoanode	16.9 10	20.8 * 13.4 *		1 h 102 h	

* Fehr et al. [15] reported STH efficiencies assuming a 100% Faradaic efficiency for the catalysts, which corresponds to an ABPE.

## Data Availability

The data presented in this study are available on request from the corresponding author.

## Supplementary Materials

The following supporting information can be downloaded at: https://www.mdpi.com/article/10.3390/mi17040431/s1, Video S1: One Nickel foil and PSC immersed; Video S2: Two Nickel foils and PSC immersed.

## Abbreviations

The following abbreviations are used in this manuscript:

OS	Outside
IS	Immersed
OER	Oxygen Evolution Reaction
HER	Hydrogen Evolution Reaction
ABPE	Applied Bias Photon-to-Current Efficiency
RHE	Reversible Hydrogen Electrode
PET–EVA	poly (ethylene terephthalate)–ethylene vinylacetate
AM	Air Mass
PSC	Perovskite Solar Cell
PEC	Photoelectrochemical
STH	Solar-to-Hydrogen
CV	Cyclic Voltammetry
ITO	Indium tin oxide
n-i-p	negative-intrinsic-positive
ETL	Electron Transport Layer
HTL	Hole Transport Layer
J–V	Current Density–Voltage
LED	Light Emitting Diode
J_sc_	Short-Circuit Current Density
V_oc_	Open-Circuit Voltage
FF	Fill Factor
PCE	Power Conversion Efficiency
J_ph_	Photocurrent Density
PV	Photovoltaic
*V*_opt_	Optimal Bias

## References

[1] NREL Best Research-Cell Efficiency Chart. https://www.nrel.gov/pv/cell-efficiency.html.

[2] Green M., Ho-Baillie A., Snaith H. (2014). The emergence of perovskite solar cells. Nat. Photon..

[3] Park N.-G., Zhu K. (2020). Scalable fabrication and coating methods for perovskite solar cells and solar modules. Nat. Rev. Mater..

[4] Niu G., Li W., Li J., Liang X., Wang L. (2017). Enhancement of thermal stability for perovskite solar cells through cesium doping. RSC Adv..

[5] Jeong M., Choi I.W., Go E.M., Cho Y., Kim M., Lee B., Jeong S., Jo Y., Choi H.W., Lee J. (2020). Stable perovskite solar cells with efficiency exceeding 24.8% and 0.3-V voltage loss. Science.

[6] Ahn N., Son D.Y., Jang I.H., Kang S.M., Choi M., Park N.-G. (2015). Highly reproducible perovskite solar cells with average efficiency of 18.3% and best efficiency of 19.7% fabricated via Lewis base adduct of lead(II) iodide. J. Am. Chem. Soc..

[7] Yang Y., Song X., Liu H., Zhang A., Cao J., Wu C. (2025). Encapsulation-driven stability in perovskite solar cells: Suppressing degradation through hermetic sealing. ACS Appl. Mater. Interfaces.

[8] Aitola K., Gava Sonai G., Markkanen M., Kaschuk J.J., Hou X., Miettunen K., Lund P.D. (2022). Encapsulation of commercial and emerging solar cells with focus on perovskite solar cells. Sol. Energy.

[9] Dipta S.S., Rahim M.A., Uddin A. (2024). Encapsulating perovskite solar cells for long-term stability and prevention of lead toxicity. Appl. Phys. Rev..

[10] Chu Q.-Q., Sun Z., Wang D., Cheng B., Wang H., Wong C.-P., Fang B. (2023). Encapsulation: The path to commercialization of stable perovskite solar cells. Matter.

[11] Walter M.G., Warren E.L., McKone J.R., Boettcher S.W., Mi Q., Santori E.A., Lewis N.S. (2010). Solar Water Splitting Cells. Chem. Rev..

[12] Qiu Y., Liu W., Chen W., Zhou G., Hsu P.-C., Zhang R., Liang Z., Fan S., Zhang Y., Cui Y. (2016). Efficient solar-driven water splitting by nanocone BiVO_4_–perovskite tandem cells. Sci. Adv..

[13] Hansora D., Yoo J.W., Mehrotra R., Byun W.J., Lim D., Kim Y.K., Noh E., Lim H., Jang J.W., Seok S.I. (2024). All-perovskite-based unassisted photoelectrochemical water splitting system for efficient, stable and scalable solar hydrogen production. Nat. Energy.

[14] Yang H., Liu Y., Ding Y., Li F., Wang L., Cai B., Zhang F., Liu T., Boschloo G., Johansson E.M.J. (2023). Monolithic FAPbBr_3_ photoanode for photoelectrochemical water oxidation with low onset-potential and enhanced stability. Nat. Commun..

[15] Fehr A.M.K., Agrawal A., Mandani F., Conrad C.L., Jiang Q., Park S.Y., Alley O., Li B., Sidhik S., Metcalf I. (2023). Integrated halide perovskite photoelectrochemical cells with solar-driven water-splitting efficiency of 20.8%. Nat. Commun..

[16] Song Z., Li C., Chen L., Dolia K., Fu S., Sun N., Li Y., Wyatt K., Young J.L., Deutsch T.G. (2023). All-perovskite tandem photoelectrodes for unassisted solar hydrogen production. ACS Energy Lett..

[17] Saliba M., Matsui T., Seo J.Y., Domanski K., Correa-Baena J.P., Nazeeruddin M.K., Zakeeruddin S.M., Tress W., Abate A., Hagfeldt A. (2016). Cesium-containing triple cation perovskite solar cells: Improved stability, reproducibility and high efficiency. Energy Environ. Sci..

[18] La Ferrara V., De Maria A., Rametta G. (2024). Green anisole as antisolvent in planar triple-cation perovskite solar cells with varying cesium concentrations. Micromachines.

[19] Garcia A.C., Koper M.T.M. (2018). Effect of saturating the electrolyte with oxygen on the activity for the oxygen evolution reaction. ACS Catal..

[20] Li W., Bao X., Zhu A., Gu H., Mao Y., Wang B., Wang G., Guo J., Li Y., Xing G. (2025). Internal Encapsulation Enables Efficient and Stable Perovskite Solar Cells. Adv. Funct. Mater..

[21] Tress W., Marinova N., Inganäs O., Nazeeruddin M.K., Zakeeruddin S.M., Grätzel M. (2015). Understanding the rate-dependent J–V hysteresis, slow time component, and aging in CH_3_NH_3_PbI_3_ perovskite solar cells. Energy Environ. Sci..

[22] Ocaña L., Montes C., González-Díaz B., González-Pérez S., Llarena E. (2024). Evaluation of ethylene-vinyl acetate, methyl methacrylate, and polyvinylidene fluoride as encapsulating materials for perovskite-based solar cells using the low-temperature encapsulation method in a cleanroom environment. Energies.

[23] Emery Q., Dagault L., Khenkin M., Kyranaki N., Bernardes de Araújo W.M., Erdil U., Demuylder M., Cros S., Schlatmann R., Stannowski B. (2025). Tips and tricks for a good encapsulation for perovskite-based solar cells. Prog. Photovolt. Res. Appl..

